# Anti-HIV Drugs Cause Mitochondrial Dysfunction in Monocyte-Derived Macrophages

**DOI:** 10.1128/aac.01941-21

**Published:** 2022-03-16

**Authors:** Jennillee Wallace, Hemil Gonzalez, Reshma Rajan, Srinivas D. Narasipura, Amber K. Virdi, Arnold Z. Olali, Ankur Naqib, Zarema Arbieva, Mark Maienschein-Cline, Lena Al-Harthi

**Affiliations:** a Department of Microbial Pathogens and Immunity, Rush University Medical Center, Chicago, Illinois, USA; b Department of Internal Medicine, Division of Infectious Diseases, Rush Medical College, Chicago, Illinois, USA; c Rush Research Informatics Core, Rush University Medical Center, Chicago, Illinois, USA; d Research Informatics Core, Research Resources Center, University of Illinois at Chicago, Chicago, Illinois, USA; e Genome Research Core, University of Illinois at Chicago, Chicago, Illinois, USA

**Keywords:** antiretroviral agents, human immunodeficiency virus, macrophages, mitochondria, monocyte-derived macrophage

## Abstract

Combination antiretroviral therapy (cART) dramatically changed the face of the HIV/AIDS pandemic, making it one of the most prominent medical breakthroughs of the past 3 decades. However, as the life span of persons living with HIV (PLWH) continues to approach that of the general population, the same cannot be said regarding their quality of life. PLWH are affected by comorbid conditions such as high blood pressure, diabetes, and neurocognitive impairment at a higher rate and increased severity than their age-matched counterparts. PLWH also have higher levels of inflammation, the drivers of which are not entirely clear. As cART treatment is lifelong, we assessed here the effects of cART, independent of HIV, on primary human monocyte-derived macrophages (MDMs). MDMs were unskewed or skewed to an alternative phenotype and treated with Atripla or Triumeq, two first-line cART treatments. We report that Triumeq skewed alternative MDMs toward an inflammatory nonsenescent phenotype. Both Atripla and Triumeq caused mitochondrial dysfunction, specifically efavirenz and abacavir. Additionally, transcriptome sequencing (RNA-seq) demonstrated that both Atripla and Triumeq caused differential regulation of genes involved in immune regulation and cell cycle and DNA repair. Collectively, our data demonstrate that cART, independent of HIV, alters the MDM phenotype. This suggests that cART may contribute to cell dysregulation in PLWH that subsequently results in increased susceptibility to comorbidities.

## INTRODUCTION

Among the most successful treatment interventions of the past 30 years, HIV antiretrovirals (ARV), particularly combination antiretroviral therapy (cART), have transformed HIV infection from a death sentence to a manageable chronic disease. cART is a combination of three antiretroviral agents, two nucleoside reverse transcriptase inhibitors (NRTIs) plus one nonnucleoside reverse transcriptase inhibitor (NNRTI) or two NRTIs plus one integrase strand transfer inhibitors (INSTI). People living with HIV (PLWH) under cART are living longer, and their life expectancy is beginning to approach that of the general population (1). In spite of these medical advances, PLWH are impacted by a growing number of comorbid conditions, most prominently, cardiovascular, metabolic, and neurocognitive dysfunction (2, 3). The mechanism(s) driving these comorbidities is not entirely clear, although persistent immune activation, even in the setting of maximum viral suppression, is thought to be a contributing factor (4). While the idea of persistent immune activation as a direct result of the HIV reservoir is plausible, limited studies have evaluated the direct impact of cART on persistent immune activation and inflammation.

Macrophages are long-lived tissue cells capable of fully orchestrating an immune response to “danger” signals. They are of the myeloid lineage and reside in all tissues. Tissue macrophages share characteristics such as plasticity and numerous immune and tissue remodeling functions, which are dictated by signals in their environments. Of interest are resident brain macrophages, which are derived either from the yolk sac during development or through monocyte transmigration into the brain and differentiation into monocyte-derived macrophages (MDMs) (5). MDM phenotypes depend on the tissue microenvironment (6, 7); they are plastic and can display phenotypes ranging from inflammatory (M1) to various alternative phenotypes with anti-inflammatory and homeostatic functions (M2s) (7, 8). Each phenotype is characterized by distinct cell surface markers, secreted cytokines, chemokines, growth factors, as well as functional capacity (7). Some phenotypes can be generated *in vitro*, such as the classical M1-MDMs induced by interferon gamma (IFN-γ) and lipopolysaccharide (LPS), or the alternative M2a-MDMs induced by interleukin 4 (IL-4) and/or IL-13 (9).

In addition, skewing of macrophages is likely to have a significant impact on their functionality in various tissues. In the context of HIV infection, macrophages in the central nervous system (CNS) are implicated in the complex combination of factors contributing to HIV-associated neurocognitive disorders (HAND) (10–14). HAND describes a spectrum of conditions that include asymptomatic neurocognitive impairment (ANI), mild neurocognitive disorder (MND), and the more severe HIV-associated dementia (HAD) (15, 16). Most of the pathogenic contribution of macrophages to HAND is described by studying cells that are infected with HIV, while the direct role of cART itself, independent of HIV infection of macrophages, is less clear.

ARVs penetrate the CNS, albeit in various degrees (17), achieving viral suppression and improvement in cognitive score in the majority of patients (18–20). Nevertheless, a growing body of evidence demonstrates that some ARVs are neurotoxic (21–23). The nonnucleoside reverse transcriptase inhibitor (NNRTI) efavirenz (EFV) causes neuronal mitochondrial toxicity (22–26). The nucleoside reverse transcriptase inhibitors (NRTIs) zidovudine (AZT), abacavir (ABC), and lamivudine (3TC) and the protease inhibitor indinavir (IDV) are associated with increased neuronal beta-amyloid production and decreased ability of microglial cells to phagocytose beta-amyloid (27). Additionally, ARV-induced CNS toxicity is augmented by aging (28).

We assessed here the impact of current cART regimens on the phenotype and function of primary human MDMs *in vitro*. We targeted current cART regimens, Atripla and Triumeq. Both are currently widely prescribed cART regimens globally. Atripla consists of emtricitabine (FTC), tenofovir disoproxil fumarate (TDF), both NRTIs, and efavirenz (EFV), an NNRTI. Triumeq consists of the two NRTIs abacavir (ABC) and lamivudine (3TC) and the integrase inhibitor dolutegravir (DTG). We observed that Triumeq-treated MDMs were skewed toward a more inflammatory phenotype with increased production of both reactive oxygen species (ROS) and inflammatory cytokines. Atripla and Triumeq also caused mitochondrial dysfunction in MDMs, and transcriptome sequencing (RNA-seq) revealed that both drugs caused the dysregulation of several genes, with Triumeq having the more significant impact. Our findings emphasize that cART impacts the phenotype of MDMs and causes mitochondrial dysfunction, which underscores an impact of cART on MDMs independent of HIV infection that is likely a contributing factor to comorbid conditions among PLWH.

## RESULTS

### Atripla and Triumeq alter MDM morphology and increase the number of cells in the G_2_/M phase.

**FIG 1:**
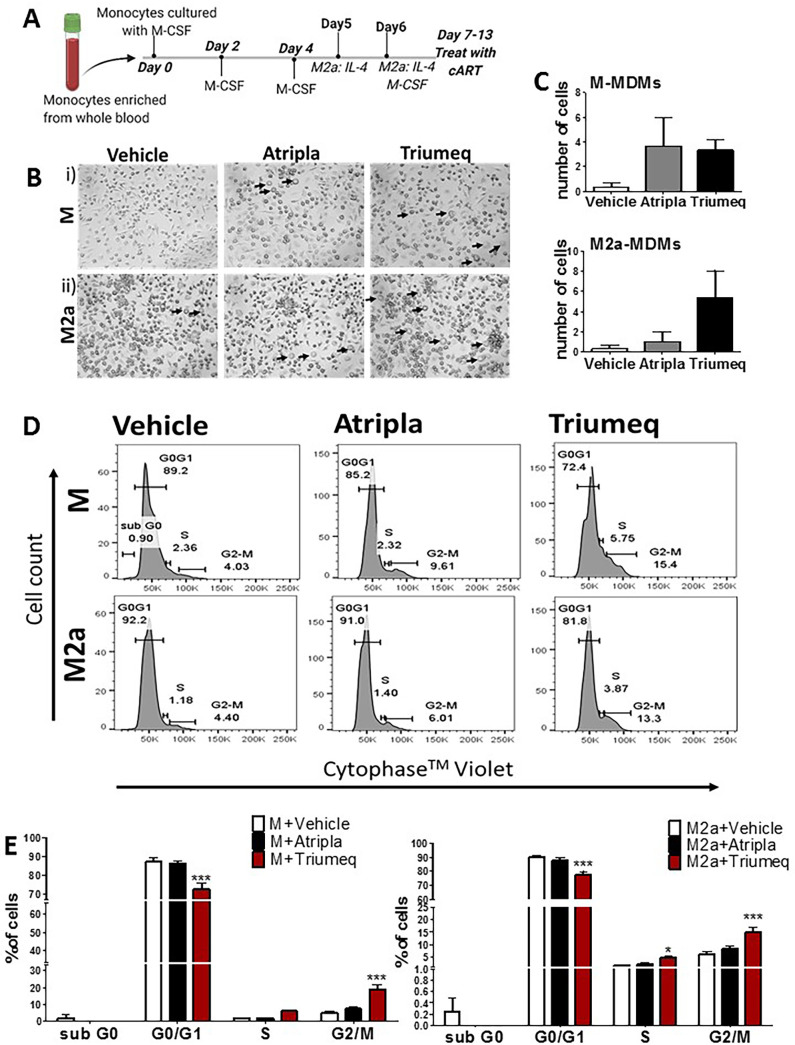
cART effect on MDM morphology and cell cycle. (A) Experimental setup and timeline of monocyte maturation into monocyte-derived macrophages and treatment with cART. (B) Brightfield microscopy at ×20 magnification taken after MDM maturation and subsequent treatment with Atripla or Triumeq. Arrows indicate large circular cells. (C) Quantification of panel B displaying average count of large circular cells observed in each treatment group, i.e., vehicle (white bars), Atripla (gray bars), or Triumeq (black bars); *n* = 3. (D) cART-treated MDMs were labeled with Cytophase Violet, a cell-permeable DNA-binding dye. Signal was measured via flow cytometry using the violet laser. Each peak represents different stages of the cell cycle, namely, G_0_, G_0_/G_1_, S, and G_2_M phases. The numbers associated with each cell cycle stage are the percentage of cells within each stage. The percentage of cells for each peak was determined using FlowJo software. (E) Representative bar graph displaying the average percentage of cells in each stage of the cell cycle displayed in panel D from four donors for both MDM phenotypes treated with vehicle or cART. M/M2a-MDMs with vehicle, white bars; (M/M2a-MDMs with Atripla, black bars); M/M2a-MDMs with Triumeq, red bars. MDM, monocyte-derived macrophage; cART, combination antiretroviral therapy; G, growth; S, DNA synthesis; M, mitosis. Two-way ANOVA; *n* = 4; *, *P* < 0.05; ***, *P* < 0.001.

Primary human MDMs were generated *in vitro* from blood monocytes and differentiated to M- or M2a-MDMs then treated every day for 1 week with Atripla or Triumeq at doses indicated in Materials and Methods (Fig. 1A). M-MDMs and M2a-MDMs treated with Atripla or Triumeq displayed increased frequency of circular adherent cells throughout the culture duration (Fig. 1B and C). To determine whether cART affected apoptosis or cell proliferation, we stained MDMs with a DNA binding dye; apoptosis and cell proliferation were not impacted in either treatment arm (Fig. 1D and E; Fig. S1 in the supplemental material); however, Triumeq resulted in an ∼10% reduction of cells in the G_0_/G_1_ phase of the cell cycle (*P* < 0.001 for M and M2a-MDMs) and a compensatory increase in the G_2_/M cell cycle stage (*P* < 0.001 for M and M2a-MDMs) (Fig. 1E). These data demonstrate that Triumeq causes cell cycle arrest in the G_2_/M phase and increases the frequency of an MDM morphology, indicative of an inflammatory phenotype (29–32).

### cART promotes an inflammatory phenotype in MDMs.

**FIG 2:**
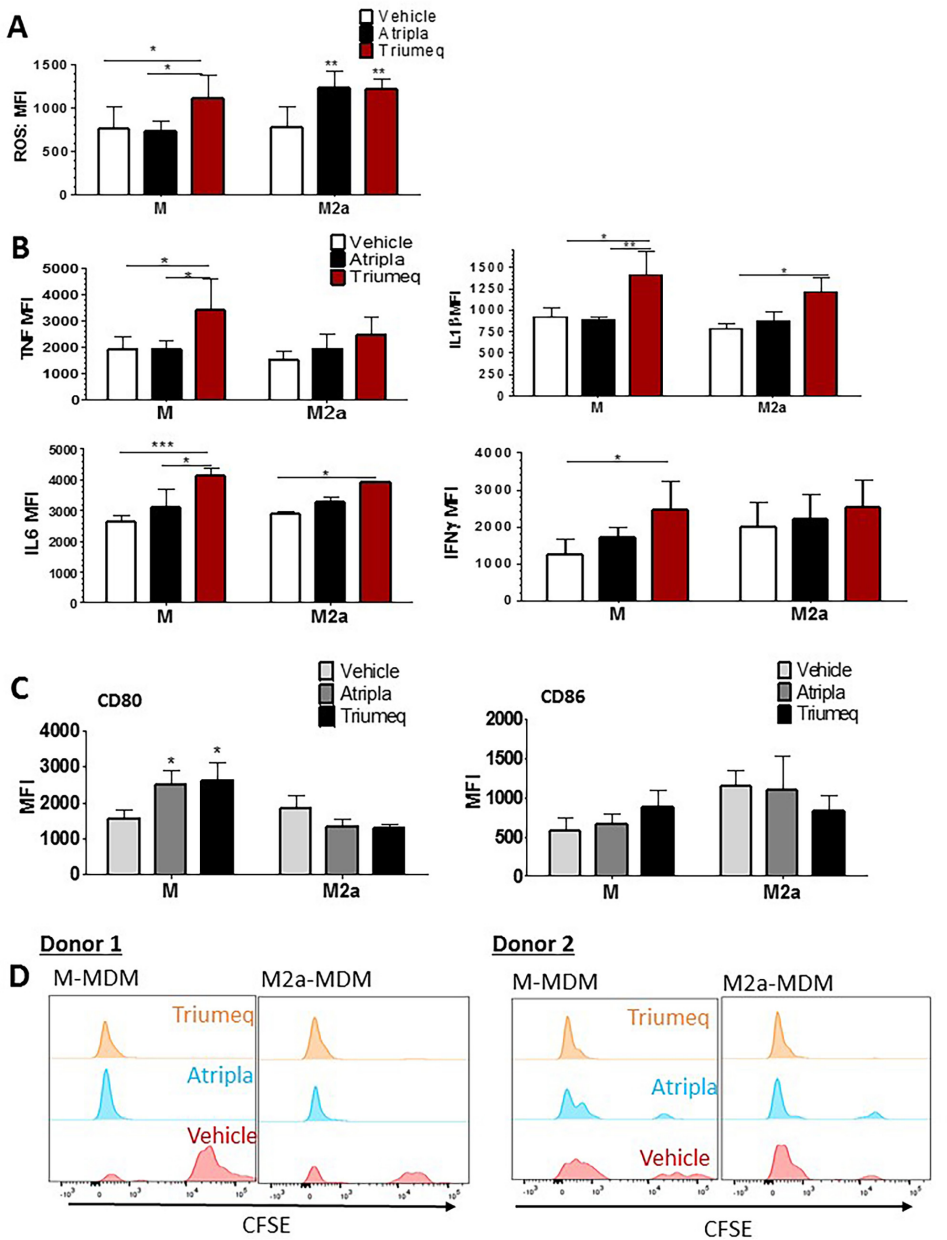
cART promotes an inflammatory phenotype in MDMs. (A) ROS production measured by flow cytometry after overnight cART treatment. (B) MDM cytokine production on the final day of cART treatment; MDMs were treated with each drug (black bars, Atripla; red bars, Triumeq) or vehicle (white bars) control in combination with brefeldin A. After 4 h, the cells were gently scraped, collected, fixed, and permeabilized to stain for TNF-α, IL-6, IL-1β, and IFN-γ production. ROS, reactive oxygen species; MFI, (geometric) mean fluorescence intensity. One-way ANOVA for M- or M2a-MDM phenotype; *n* = 3; *, *P* < 0.05; **, *P* < 0.01; ***, *P* < 0.001. (C) M- and M2a-MDMs treated with cART (medium-gray bars, Atripla; black bars, Triumeq) or vehicle control (light gray bars) were stained for costimulatory proteins CD80 and CD86 and the data represented as MFI. (D) Naive T cells were isolated and stained with CellTRace CFSE proliferation dye and then cocultured with donor-matched MDMs for 72 h. CFSE intensity was measured by flow cytometry and donor-independent cell proliferation displayed as histograms to visualize relative cell proliferation among the experimental and control groups. MDM, monocyte-derived macrophage; CFSE, carboxyfluorescein succinimidyl ester.

Because the changes in cell morphology indicated that Triumeq skewed MDMs toward an inflammatory phenotype, we measured both the production of ROS and inflammatory cytokines both in M-MDMs and the alternatively skewed M2a-MDMs. We observe that even without stimulation, Triumeq increased ROS production in M-MDMs (*P* < 0.05), and both drugs increased ROS production in the alternatively skewed M2a-MDMs (*P* < 0.01) by approximately 1.5-fold (Fig. 2A). We then measured the production of inflammatory cytokines, again with cART treatment in the absence of additional stimulation, and observed that Triumeq and not Atripla caused a significant increase in tumor necrosis factor alpha (TNF-α) in M-MDMs by approximately 1.5-fold (*P* < 0.05), in IL-1β in both M-MDMs and M2a-MDMs by approximately 1.5-fold compared to both vehicle control and the Atripla-treated M-MDMs (*P* < 0.05 and *P* > 0.01, respectively), in IL-6 in both M-MDMs and M2a-MDMs by approximately 1.5-fold (*P* < 0.001, Triumeq versus vehicle; *P* < 0.05, Triumeq- versus Atripla-treated MDMs) and 1.3-fold, respectively (*P* < 0.05), and in IFN-γ in M-MDMs by approximately 2-fold (*P* < 0.05) (Fig. 2B). Further, both Atripla and Triumeq induced a significant increase in CD80 but not CD86 expression; both are costimulatory molecules associated with the ability of MDMs to activate T cells (Fig. 2C) (33–35).

Following the observed increase in CD80 expression on MDMs, we assessed the downstream effects of these changes in MDMs on T cell proliferation in response to cART treatment. Briefly, activated naive T cells (2 donors) cocultured with cART-treated macrophages showed an increased rate of proliferation in response to αCD3/αCD28 stimulation. Activated naive T cells cocultured with Triumeq-treated M- and M2a-MDMs showed increased proliferation as determined by loss of cell trace carboxyfluorescein succinimidyl ester (CFSE) staining compared to T cells cultured with vehicle-treated M- and M2a-MDMs (Fig. 2D). Together, these findings demonstrate that both Atripla and Triumeq activate MDMs, while Triumeq skews M- and M2a-MDMs toward a more inflammatory phenotype, which can further induce proliferation of T cells.

### Triumeq increases mitochondrial membrane potential.

**FIG 3:**
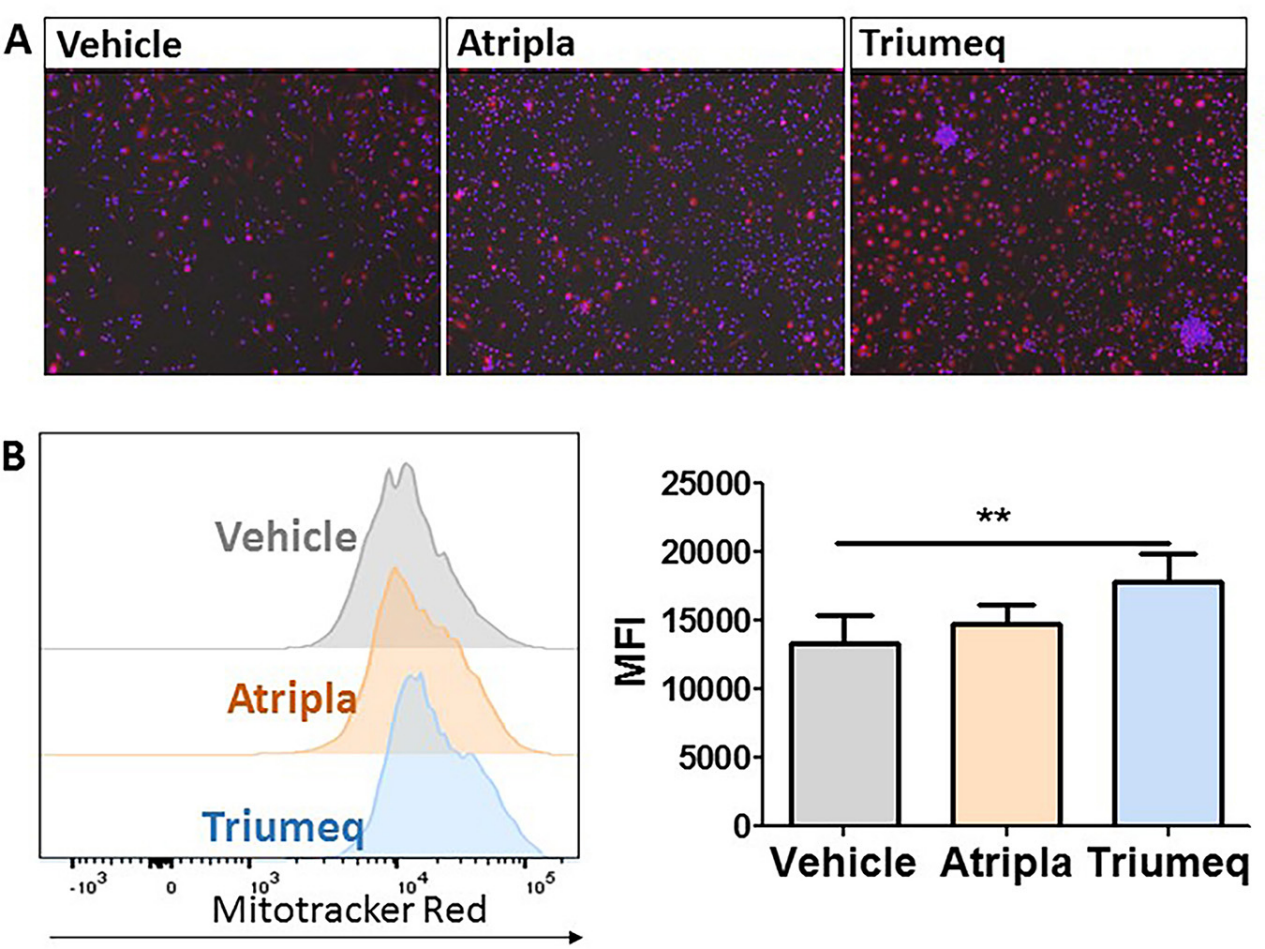
Increased MitoTracker Red. (A) Fluorescence MitoTracker Red staining in cART-treated MDMs; ×20 magnification. (B) Quantification of MitoTracker Red by flow cytometry illustrated in a representative bar graph and summary of MFIs. MFI, (geometric) mean fluorescence intensity. One-way ANOVA; *n* = 4; **, *P* < 0.01.

ROS are mainly generated inside mitochondria, and one of the key indicators of mitochondrial activity is the proton gradient which serves as an intermediate source of energy utilized by ATP synthase to make ATP (36). M-MDMs were stained with a MitoTracker Red fluorescent dye and imaged by fluorescence microscopy (Fig. 3A), and fluorescence was quantified by flow cytometry. The cumulative mean fluorescence intensity (MFI) for Triumeq-treated MDMs was significantly higher, ∼25% increase, compared to vehicle-treated M-MDMs (Fig. 3B). No significant change was observed with Atripla-treated MDMs. These data demonstrate that Triumeq, and not Atripla, increases mitochondrial membrane potential.

### cART causes mitochondrial dysfunction in MDMs relative to vehicle-treated MDMs.

**FIG 4:**
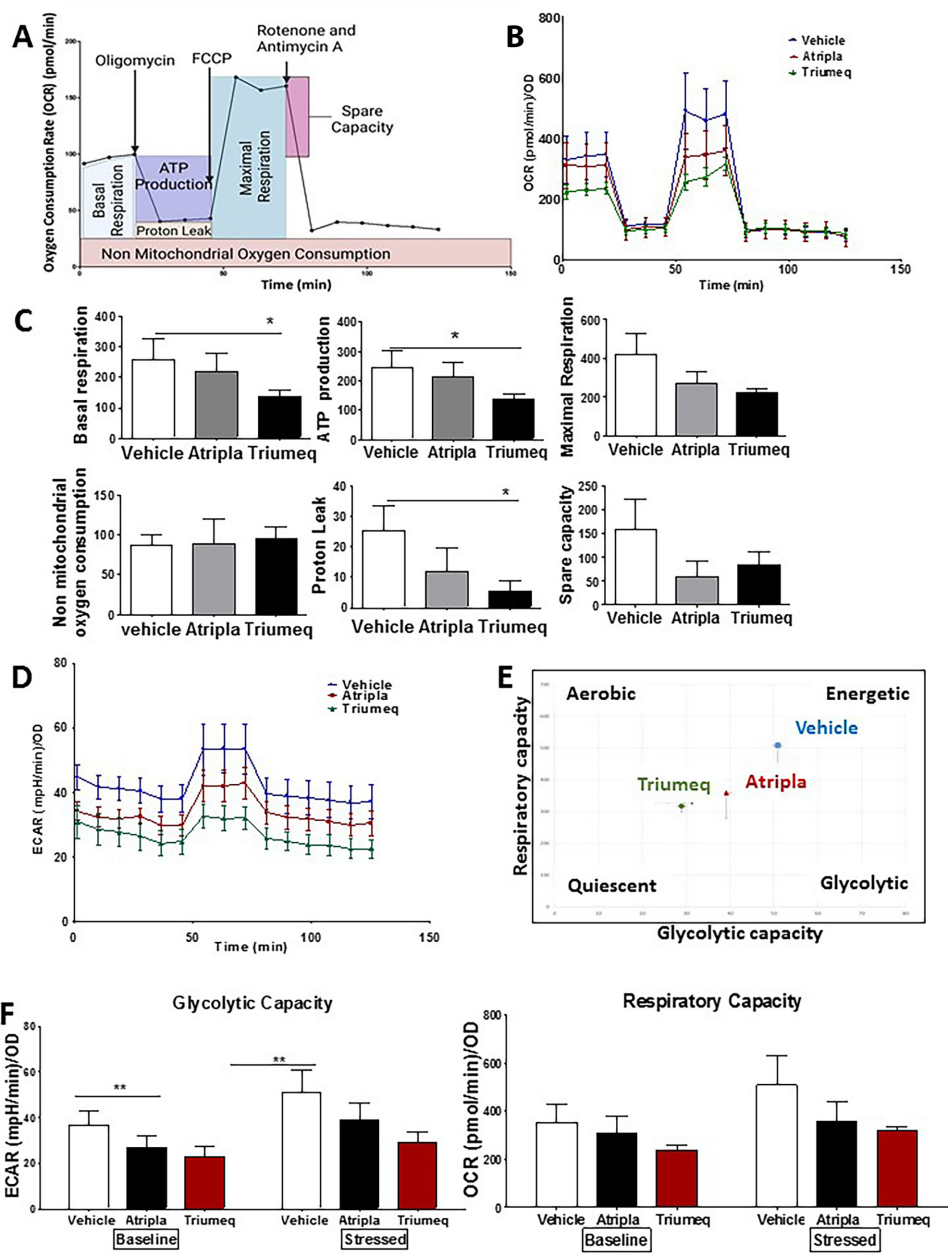
Mito stress test following treatment with cART. (A) The Mito stress test provides OCR and ECAR data by measuring several bioenergetics properties. OCR measurements are made following the addition of modulators of the electron transport chain, specifically, (i) oligomycin inhibits ATP synthase and decreases OCR. Basal respiration is measured prior to oligomycin addition, while ATP production and proton leak are assessed after the addition of oligomycin; (ii) FCCP uncouples oxygen consumption and ATP production and increases OCR, allowing for the assessment of maximal respiration; and (iii) rotenone and antimycin A inhibit complexes I and III of the electron transport chain, reducing mitochondrial ATP production and reducing OCR, allowing for the assessment of spare capacity. (B) Average OCR for MDMs treated with vehicle control (blue), Atripla (red), or Triumeq (green) normalized to total protein. Error bars demonstrate SEM. (C) Bar graphs representing changes in the different parameters measured by the Mito stress test, i.e., basal respiration, ATP production, maximal respiration, nonmitochondrial oxygen consumption, proton leak, and spare capacity. (D) Average ECAR for MDMs treated with vehicle control (blue), Atripla (red), or Triumeq (green) normalized to total protein. Error bars demonstrate SEM. (E) Energy profile of cART-treated MDMs. The *y* and *x* axes display mitochondrial respiration (OCR) and glycolytic capacity (ECAR) after the addition of oligomycin, i.e., stressed OCR and stressed ECAR. (F) Energy profile of cART-treated MDMs both at baseline before the addition of oligomycin and stressed after oligomycin addition. The *y* axis represents either glycolytic capacity (ECAR) or mitochondrial respiration (OCR) for MDMs treated with vehicle control (white bars), Atripla (black bars), or Triumeq (red bars). OCR, oxygen consumption rate; ECAR, extracellular acidification rate; OD, optical density; SEM, standard error of the mean. One-way ANOVA; *n* = 6 independent experiments; *, *P* < 0.05; **, *P* < 0.01.

We next performed a Mito stress test to address the extent of mitochondrial dysfunction under cART treatment. The Mito stress test measures several criteria (Fig. 4A), which, together, provide information on the oxygen consumption rate (OCR) and extra cellular acidification rate (ECAR) or the respiratory capacity and glycolytic capacity, respectively. To assess whether Triumeq and Atripla caused mitochondrial dysfunction in MDMs, cells were cultured as described earlier, and a Mito stress test was performed at the end of the treatment period. We observed a lower OCR in cART-treated cells (Fig. 4B); particularly, there was a significant decrease in basal respiration, ATP production, and proton leak associated with Triumeq (Fig. 4C). Additionally, ECAR was lowest in Triumeq-treated cells, which, relative to vehicle- and Atripla-treated MDMs, demonstrated the lowest glycolytic capacity and mitochondrial respiration rate (Fig. 4D and E). Baseline measurements of OCR and ECAR were lower in Triumeq-treated MDMs, with ECAR demonstrating a significant difference, and this relationship remained after the cells were stressed with oligomycin, (Fig. 4F).

**FIG 5:**
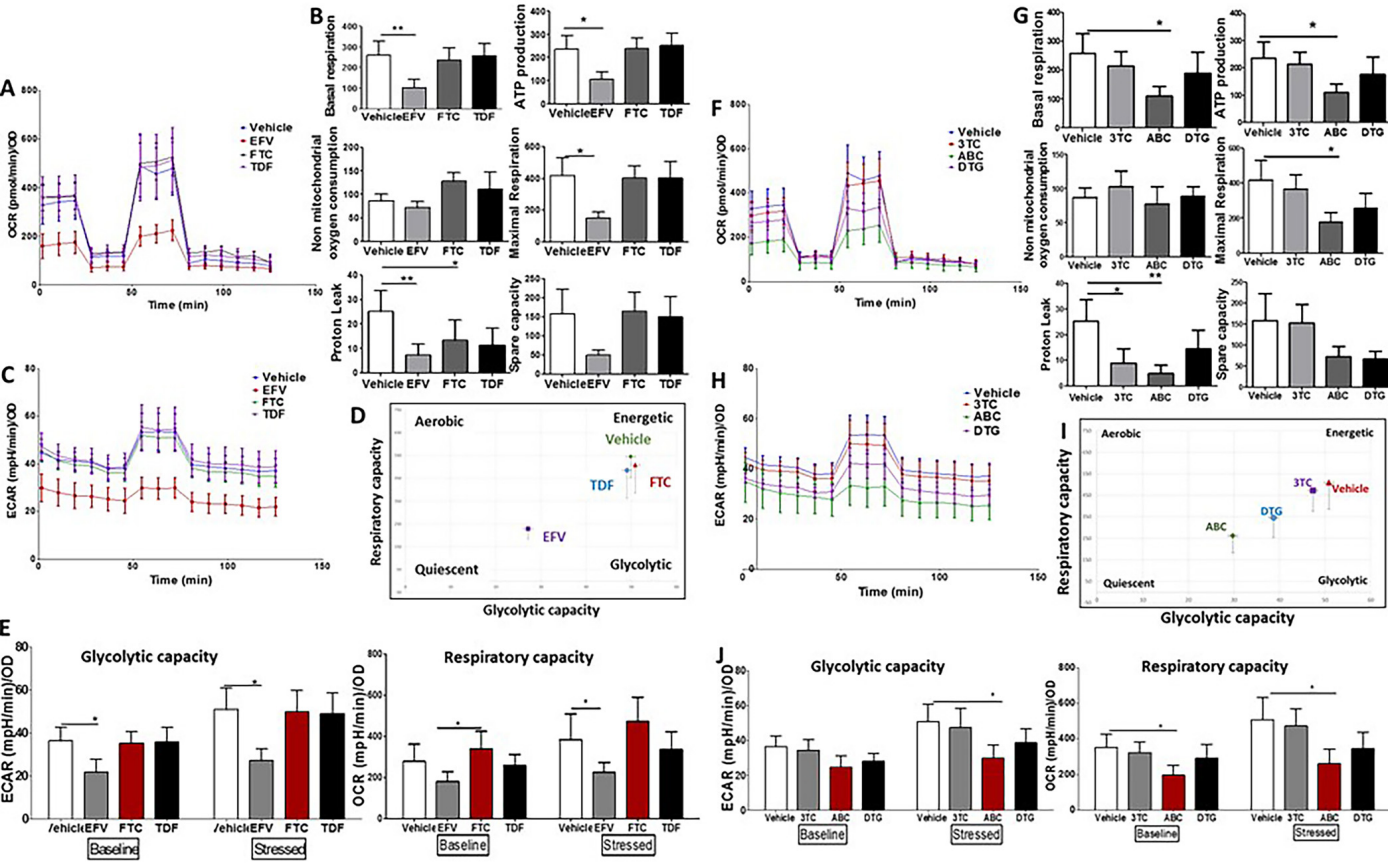
Mito stress test following treatment with individual components of cART. (A) Average OCR for MDMs treated with vehicle control (blue), EFV (red), FTC (black), or TDF (purple) normalized to total protein. Error bars demonstrate SEM. (B) Bar graphs representing changes in the different parameters measured by the Mito stress test, i.e., basal respiration, ATP production, maximal respiration, nonmitochondrial oxygen consumption, proton leak, and spare capacity. (C) Average ECAR for MDMs treated with vehicle control (blue), EFV (red), FTC (black), or TDF (purple) normalized to total protein. Error bars demonstrate SEM. (D) Energy profile of cART-treated MDMs. The *y* and *x* axes display mitochondrial respiration (OCR) and glycolytic capacity (ECAR) after the addition of oligomycin, i.e., stressed OCR and stressed ECAR. (E) Energy profile of cART-treated MDMs both at baseline before the addition of oligomycin and stressed after oligomycin addition. The *y* axis represents either glycolytic capacity (ECAR) or mitochondrial respiration (OCR) for MDMs treated with vehicle control (white bars), EFV (gray bars), FTC (red bars), or TDF (black bars). (F) Average OCR for MDMs treated with vehicle control (blue), 3TC (red), ABC (green), or DTG (purple) normalized to total protein. Error bars demonstrate SEM. (G) Bar graphs representing changes in the different parameters measured by the Mito stress test, i.e., basal respiration, ATP production, maximal respiration, nonmitochondrial oxygen consumption, proton leak, and spare capacity. (H) Average ECAR for MDMs treated with vehicle control (blue), 3TC (red), ABC (green), or DTG (purple) normalized to total protein. Error bars demonstrate SEM. (I) Energy profile of cART-treated MDMs. The *y* and *x* axes display mitochondrial respiration (OCR) and glycolytic capacity (ECAR) after the addition of oligomycin, i.e., stressed OCR and stressed ECAR. (J) Energy profile of cART-treated MDMs both at baseline before the addition of oligomycin and stressed after oligomycin addition. The *y* axis represents either glycolytic capacity (ECAR) or mitochondrial respiration (OCR) for MDMs treated with vehicle control (white bars), 3TC (gray bars), ABC (red bars), or DTG (black bars). OCR, oxygen consumption rate; ECAR, extracellular acidification rate; OD, optical density. SEM, standard error mean; EFV, efavirenz; FTC, emtricitabine; TDF, tenofovir disoproxil fumarate; 3TC, lamivudine; ABC, abacavir; DTG, dolutegravir. One-way ANOVA; *n* = 6; *, *P* < 0.05; **, *P* < 0.01.

Next, we determined whether an individual component of each cART was responsible for the mitochondrial dysfunction observed. MDMs were treated with the individual components of both Atripla and Triumeq for a week after MDM differentiation. Of the individual components of Atripla (i.e., FTC, TDF, and EFV), EFV-treated MDMs demonstrated significant reduction in OCR (Fig. 5A). Of the parameters assessed, EFV treated-MDMs showed a significant decrease in basal respiration, ATP production, maximal respiration, and proton leak; neither FTC nor TDF had these significant effects (Fig. 5B). EFV also demonstrated the lowest ECAR relative to vehicle-, FTC-, or TDF-treated MDMs (Fig. 5C) over time. Even at baseline, before cells were treated with oligomycin, EFV-treated MDMs showed the lowest glycolytic capacity and mitochondrial respiration rate (Fig. 5D and E). Of the individual components of Triumeq, ABC-treated MDMs demonstrated a significant reduction in OCR (Fig. 5F). Of the parameters assessed, ABC-treated MDMs showed a significant decrease in basal respiration, ATP production, maximal respiration, and proton leak, which was also significantly lower in 3TC-treated MDMs (Fig. 5G). While DTG also showed decreases in ECAR and OCR, the differences were not statistically significant. ECAR was lowest in ABC-treated MDMs over time (Fig. 5H), and ABC-treated MDMs also demonstrated lower baseline glycolytic capacity and mitochondrial respiration over time (Fig. 5I to J). Overall, these findings demonstrate that both Atripla and Triumeq cause mitochondrial dysfunction in primary human MDMs, with Triumeq-treated MDMs showing a significant decrease in the measured parameters of mitochondrial respiration. Of the drugs that comprise Atripla, EFV caused more dysfunction than FTC and TDF in Atripla, while ABC caused more mitochondrial dysfunction in Triumeq-treated MDMs than 3TC- and DTG-treated cells.

### cART affects gene expression in MDMs.

**FIG 6:**
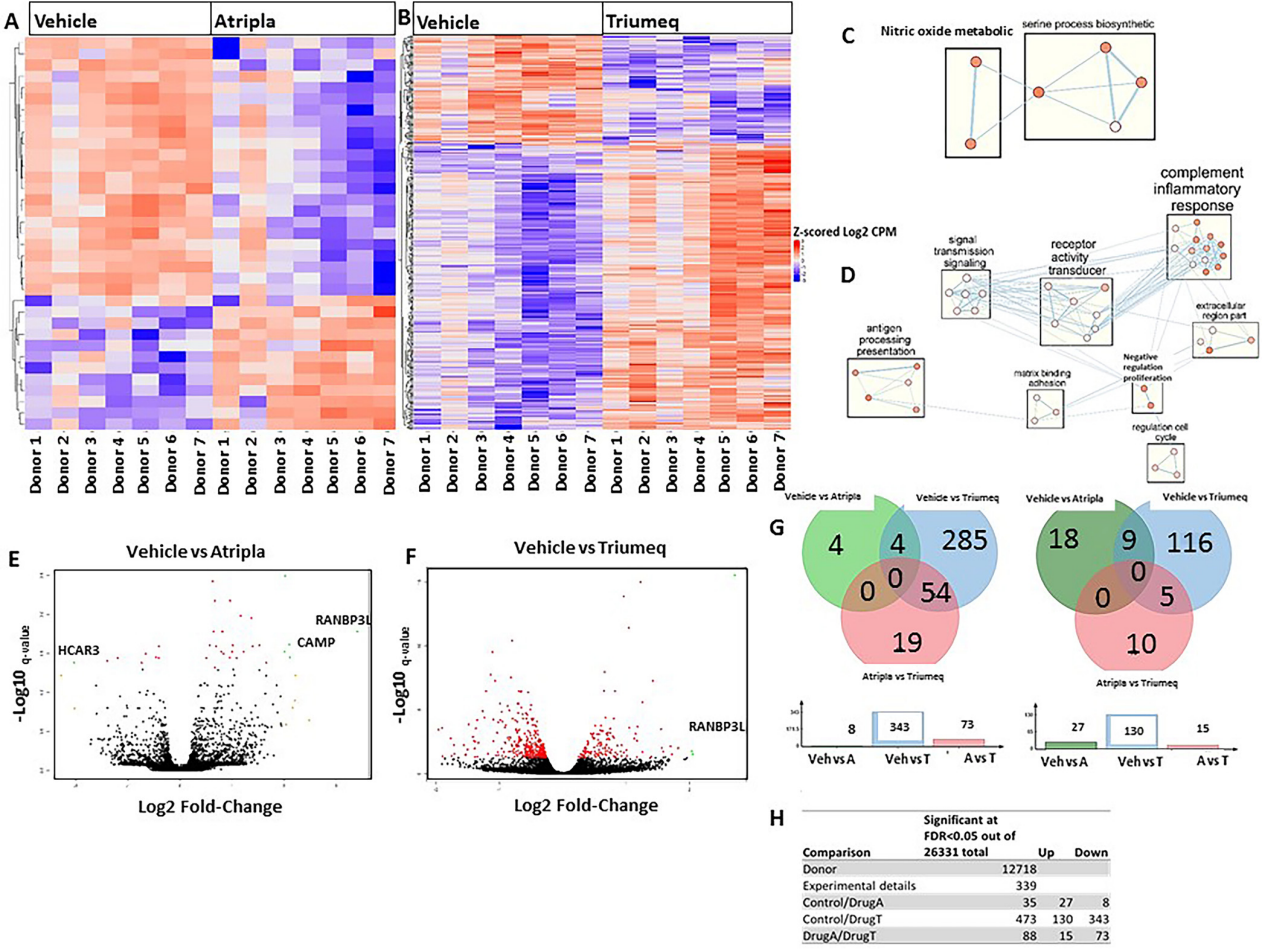
Differentially expressed genes in cART-treated M-MDMs. (A and B) Heat map of differentially expressed genes in MDMs treated with Atripla (A) or Triumeq (B); *n* = 7 donors. (*q* ≤ 0.05 for both drugs) (C and D) Enrichment pathways in both Atripla- (C) and Triumeq-treated (D) MDMs (*q* ≤ 0.05 for Atripla and *q* ≤ 0.01 for Triumeq). Clustering was performed using BiNGO. (https://pubmed.ncbi.nlm.nih.gov/15972284/) to determine the Gene Ontology (GO) categories that are statistically overrepresented in a set of genes. The thickness of the lines refers to the weighted graphs. A thicker line means a stronger relationship, and a thinner line refers to a comparatively weaker relationship. The shading refers to more genes in common between pathways. (E and F) Volcano plot illustrating the most significant upregulated genes in vehicle versus Atripla (E) or vehicle (F) versus Triumeq-treated cells. Red dots, any gene with significant *q* value less than 0.05; orange dots, absolute logFC > 2; and green dots, *q* < 0.05 and absolute logFC > 2. Genes listed on each volcano plot are those that have both a significant *q* value and absolute logFC greater than 2. (G and H) Total number of dysregulated genes.

To determine the extent to which Atripla and Triumeq impact gene expression in MDMs, RNA-seq was performed for M-MDMs from 7 donors treated with Atripla, Triumeq, or vehicle control. Results demonstrate that both drugs caused significant changes in multiple genes (Fig. 6A and B; Data Set S1) involved in pathways such as serine processing, nitric oxide metabolism (Atripla) (Fig. 6C; Data Set S3) and cell signaling; cell cycle regulation; response to stimuli; and immune regulation (Triumeq) (Fig. 6D; Data Set S3). Volcano plots demonstrated that the Ran binding protein 3-like (*RANBP3L*) expression was significantly upregulated in both Atripla- and Triumeq-treated cells, while cathelicidin antimicrobial peptide (*CAMP*) was increased in Atripla-treated cells compared to vehicle controls, and hydroxycarboxylic acid receptor 3 (*HCAR3*) expression was increased in vehicle controls compared to Atripla-treated cells (Fig. 6E and F; Data Set S2). Overall, at a false-discovery rate (*q* value) less than or equal to 0.05, there were 35 and 473 genes significantly dysregulated by Atripla and Triumeq, respectively, relative to vehicle control. In Atripla-treated cells, 27 genes were upregulated, and 8 were downregulated, while in Triumeq-treated cells, 130 genes were upregulated, and 343 were downregulated (Fig. 6G and H). These data demonstrate that cART causes dysregulation of gene expression in MDMs. Overall, we show that Atripla- and Triumeq-treated MDMs have altered immune phenotypes, including change in cytokine profile, increased ROS, and mitochondrial dysfunction. This was corroborated by RNA-seq results showing significant dysregulation of gene expression.

## DISCUSSION

The life expectancy of PLWH is close to that of the general population because of antiretroviral therapy. However, the quality of life of the HIV-positive population, even under cART, is well below that of age-matched seronegative persons (37, 38). A number of comorbid conditions exist, and though they have, in part, been attributed to low-level chronic inflammation, it is unclear what additional factors drive these comorbidities in the modern era of cART. Particularly, while significant emphasis is placed on the HIV reservoir and low-level virus production that can be mediating inflammation and contributing to HIV-associated comorbidities, the effect of cART itself independent of HIV is an understudied area. CART treatment is lifelong since cessation of cART drives robust viral rebound (39, 40). Our objective was to study the potentially toxic effects of cART at the cellular level. As such, we assessed here the impact of cART, independent of HIV, on MDMs because MDMs and tissue myeloid cells are critical cell populations that are long-lived, present in all tissues, and dysregulated in the context of HIV.

Our findings demonstrate that the phenotype of macrophages, independent of HIV, is influenced by two widely used regimens for cART, Atripla and Triumeq. Atripla is comprised of TDF, FTC and EFV, while Triumeq includes 3TC, ABC, and DTG. Specifically, Triumeq activates MDMs *in vitro*, skewing alternative MDMs toward a more inflammatory phenotype that includes increased ROS production and an increase in the inflammatory cytokines IL-1β, TNF, IFN-γ, and IL-6. Dysregulation of each of these cytokines has been linked to different neuroinflammatory disorders, including HAND. For example, IL-1β is a driver of multiple sclerosis (MS), and mice deficient in IL-1β are resistant to experimental autoimmune encephalomyelitis (EAE) (41). TNF-α secreted by glial cells generally perpetuates neuroinflammation (42), and IFN-γ activates glial cells and is increased in Parkinson’s disease (43). Finally, IL-6, a pleiotropic cytokine, has several roles that can be both neuroprotective or neuroinflammatory (44). Additionally, we observed an increase in the production of ROS and in mitochondrial membrane potential, both indicators of increased mitochondrial activity. Specifically, mitochondrial membrane potential is an indicator of the amount of stored energy used by ATP synthase for ATP production, and any prolonged decrease or increase can result in cellular toxicity (36). There was an increase in CD80 expression on M-MDMs treated with either Atripla or Triumeq resulting in increased T cell proliferation, and while there was no observed significant increase in MDM proliferation caused by cART, we did observe a significant Triumeq-related reduction of cells in the G_0_/G_1_ phase of the cell cycle and arrest in the G_2_/M cell cycle stage, a checkpoint where DNA damage is repaired (45–47). These findings are therefore suggestive of Triumeq-induced DNA damage and warrant further studies. Because of the increase in inflammatory cytokine production and observed cell cycle arrest, we evaluated the expression of senescent-associated markers p16, p53, and β-galactosidase during the culture period. However, we did not observe a significant change; indeed, β-galactosidase trended toward a decrease in expression. This finding does not negate the possibility that cART can induce senescence in MDMs since PLWH have to take cART drugs for the duration of their lives versus the *in vitro* culture period during which MDMs were assessed in our studies.

One way in which drug-induced toxicity manifests is mitochondrial dysfunction (48, 49). In fact, a Mito stress test demonstrated increased mitochondrial dysfunction in MDMs treated with both Atripla and Triumeq. The Seahorse XFe extracellular flux analyzers and Mito stress test kit allow for the measurement of the oxygen consumption rate (OCR) as well as the extracellular acidification rate (ECAR) of cells treated with cART. Specifically, the assay achieves this by monitoring both oxygen concentration and pH changes in the cell culture media over time in response to treatment with modulators of the electron transport chain that interrupt ATP production (50). These measurements allow for the indirect assessment of respiration-linked ATP production and require that the cells are in an even monolayer without clustering, which could affect the assay results. Further, use of the modulators can themselves cause cell toxicity, which is why it is important to normalize the assay results to criteria such as live cell count or total protein after the assay is complete. Nevertheless, the Seahorse extracellular flux analyzer is a valuable tool, particularly for adherent cells in culture, to advise more in-depth exploration of mitochondrial dysfunction.

We observed that both Atripla and Triumeq caused decreased OCR and ECAR in MDMs. Further, when broken down to the individual components of both cART regimens, we observed that EFV of Atripla and ABC of Triumeq showed significant mitochondrial dysfunction. Triumeq demonstrated significantly lower basal respiration, ATP production, and proton leak relative to vehicle control cells. Also at baseline, prior to addition of oligomycin, Triumeq-treated cells had lower glycolytic capacity, which was maintained after addition of oligomycin, an inhibitor of ATP synthase, that decreases OCR and increases ECAR to a maximum (50, 51). The second modulator added to the cells, carbonyl cyanide 4-(trifluoromethoxy) phenylhydrazone (FCCP), uncouples oxygen consumption and ATP production and increases OCR, while rotenone and antimycin A inhibit complexes I and III of the electron transport chain, reducing mitochondrial ATP production and reducing OCR. The addition of each modulator allowed for the measurement of key parameters of the electron transport chain (50–52). When we assessed the individual components of each drug to determine which antiretroviral was most responsible for the mitochondrial dysfunction, we observed that EFV (Atripla) caused a significant decrease in basal respiration, ATP production, maximal respiration, and proton leak. Basal respiration is derived from the baseline OCR minus nonmitochondrial respiration; therefore, basal respiration and baseline, which EFV decreased compared to vehicle control, but not significantly, are not the same values. Similar to Triumeq, ABC decreased basal respiration, ATP production, and maximal respiration, and both ABC and 3TC decreased proton leak. Neither FTC, TDF, nor DTG caused significant mitochondrial dysfunction individually in MDMs *in vitro*. Several studies have demonstrated mitochondrial toxicity induced by EFV (53–56), and our studies confirmed these effects in macrophages, a much more ubiquitous cell type than has been assessed previously. Notably, EFV was less toxic to mitochondria in the presence of FTC and TDF, suggesting a drug antagonism that potentially reduces EFV toxicity, but this notion requires further investigation. Further, ECAR, which indicates glycolytic capacity, was significantly lower for Triumeq and EFV. Studies have shown that glycolytic capacity is associated with cell damage, as a decrease is associated with hyperoxia (57), Conversely, an increased glycolytic capacity is associated with cellular reprogramming (58, 59). Macrophages are known to be plastic, an important feature needed to execute their various functions; therefore, whether cART affects macrophage ability to be reprogrammed in response to biological signals should be assessed in future studies. EFV has been a first-line choice for decades because of its efficacy; this has led to the creation of generic, and therefore lower-cost, forms, which is important in developing countries where the majority of HIV cases are found. Unfortunately, there is also greater exposure to other microbes in developing countries, and an impaired innate immune response will make HIV-positive patients on MDM-toxic cART more susceptible to other infections. Similarly, the diminished capacity of MDMs to respond to stress can be linked to an increased susceptibility to diabetes where MDMs and tissue macrophages cause chronic inflammation and display impaired wound healing capability (60, 61).

Finally, via RNA-seq, we show that several signaling pathways were influenced primarily by Triumeq, including immune regulation, cell cycle, and DNA repair, which is in agreement with the demonstrated findings of this study. Our selection criteria revealed 473 dysregulated genes in Triumeq-treated MDMs compared to 35 dysregulated genes in Atripla-treated cells. Notably, the gene *HCAR3* was upregulated in vehicle-treated cells compared to Atripla controls, while in Atripla-treated cells, *CAMP* was upregulated relative to vehicle-treated cells, and *HCAR3* is reported to regulate *CAMP* expression (62). Importantly, Triumeq affected more genes and pathways than Atripla, with minimal overlap. Considering both combinations consist of two NRTIs, it is not certain why one cART would have such a different effect with minimal overlap from the other. However, it is known that even antiretroviral drugs of the same class have their own unique side effects (63). One could speculate that this is based on structural differences since even subtle changes in the chemical structure of a drug can significantly affect their potential toxicity (64, 65). Pertaining to our observations, the relatively smaller cytidine analogs 3TC and FTC had noticeably less effect on mitochondrial dysfunction than the larger guanosine analog ABC. This further underscores the importance of studying cellular toxicity induced by antiretrovirals both individually and in the combinations in which they are administered since our data suggest that no two drugs from the same class, particularly NRTIs (ABC, FTC, 3TC, and TDF) were alike in the genes that they dysregulated in MDMs. Additionally, both the INSTI (DTG) and the NNRTI (EFV), present in Triumeq and Atripla, respectively, could contribute to the minimal overlap in dysregulated genes since these are from two different drug classes.

Drug toxicity is a common phenomenon for HIV in both the pre- and post-cART eras. Following the initiation of monotherapy with azidothymidine (AZT), the first NRTI used in human subjects, reports of toxicity soon followed (66). The most prominent complications included bone marrow toxicity and peripheral neuropathy. By the time combination therapy was developed, NRTIs were known to cause prominent mitochondrial toxicity through the inhibition of mitochondrial DNA polymerase gamma, which is required for replication of mitochondrial DNA (mtDNA) (67). More recent investigations have implicated the production of ROS as an alternative mechanism of mitochondrial toxicity (68). NRTIs like stavudine (d4T) and didanosine (ddI) have been phased out of the market due to prominent toxicities, principally lipodystrophy and peripheral neuropathy (69, 70). Gene expression profile studies have shown that cART has an additive effect on mitochondrial dysfunction compared to monotherapy (71). Furthermore, duration of use correlates with accumulation of cART-induced mitochondrial damage (72), which, in turn, is related to slow clearance of nucleoside analogs once integrated into mitochondrial DNA (73–75). This takes prominence when considering that PLWH will be exposed to cART for life.

As they pertain to the central nervous system (CNS), a number of investigators have described the penetration and accumulation of ARV in this compartment. Letendre et al. have developed the CNS penetration effectiveness (CPE) rank to classify these agents (17). cART-induced neuronal toxicity was first described by Robertson et al. by documenting reduction in MAP-2 *in vitro* (76). Whereas previous studies have used supratherapeutic or wide-ranging drug concentrations, we designed our studies with CNS-relevant concentrations in mind; this increases the accuracy and relevance of our results. Particularly, concentrations of each drug previously reported in the cerebrospinal fluid (CSF) were used (77–82). Myeloid cells in HAND are not only known sources of HIV productive infection and the HIV reservoir, but the susceptibility of myeloid cells to environmental stimuli and their subsequent influence on neighboring cell types implicate CNS macrophages as key drivers of HAND.

In HIV, a balance should be struck between the benefits of cART in suppressing HIV replication and a regimen that has none to minimum cytotoxicity, including perturbations at molecular and cellular levels. Understating the effects of anti-HIV drugs at the molecular and cellular levels will guide point-of-care decisions regarding choice of anti-HIV drugs and development of drugs with none to minimal effects on key cell populations in immunity. Further, understating the impact of anti-HIV drugs, independent of HIV, can decipher the role of the virus itself on dysregulation of cells versus that mediated by long-term use of anti-HIV drugs.

## MATERIALS AND METHODS

### Ethics statement.

Research involving human subjects was conducted in accordance with institutional (RFA-MH-20-115) and U.S. government guidelines on human research. Whole blood was collected from healthy seronegative donors at Rush University Medical Center, and all donors signed informed consents prior to donating.

### Antiretroviral drugs.

Drugs were obtained from the NIH AIDS reagent program (tenofovir disoproxil fumarate [TDF], catalog no. 10198; emtricitabine [FTC], catalog no.10071; lamivudine [3TC], catalog no. 8146; abacavir [ABC], catalog no. 4680; and efavirenz [EFV], catalog no. 4624) and Alsachim (DTG, catalog no. C4672). Drug combinations consisted of TDF, FTC, and EFV (Atripla) and ABC, 3TC, and dolutegravir (DTG) (Triumeq). The concentrations used were 0.01 μg/mL TDF (77), 0.08 μg/mL FTC (78), 0.02 μg/mL EFV (79), 2 μg/mL ABC (80), 0.32 μg/mL 3TC (81), and 0.02 μg/mL DTG (82). Both dimethyl sulfoxide (DMSO) (EFV, DTG) and water (TDF, FTC, ABC, and 3TC) were used as vehicle.

### BCA assay for total protein.

Cells were lysed with radioimmunoprecipitation assay (RIPA) buffer for bicinchoninic acid (BCA) assay following the manufacturer’s instructions. Briefly, cell lysates were loaded into 96-well assay plates in duplicates with assay reagents A and B and incubated for 30 min at 37°C with 5% CO_2_. Optical density was then measured at 450 nm (OD_450_) using a microplate reader.

### Generation of MDMs.

Primary human monocytes were isolated from whole blood of healthy donors using the RosetteSep human monocyte enrichment cocktail (StemCell Technologies, Vancouver, Canada) per the manufacturer’s protocol. Freshly isolated monocytes were cultured in a 12-well plate at a density of 100,000 to 300,000 cells/mL in complete RPMI (cRPMI) with 10% fetal bovine serum (Corning, Corning, NY), 1% penicillin and streptomycin, 1% l-glutamine, and 50 ng/mL macrophage colony-stimulating factor (M-CSF) (R&D Systems, Minneapolis, MN) at 37°C and 5% CO_2_ incubator for 5 days. M-CSF was added every other day. On day 6, cells were skewed to an alternative M2a phenotype by treating with 10 ng/mL IL-4 (Abcam, Cambridge) on both days 6 and 7 (83). Treatment with cART, either Atripla or Triumeq, or vehicle control commenced on day 8 and continued daily for an additional 7 days.

### Cell surface and intracellular immunostaining and flow cytometry.

MDMs were collected by gentle scraping, washed with Dulbecco’s phosphate-buffered saline (DPBS) (Corning, Corning, NY, USA), and incubated with Aqua live/dead fixable cell stain (Thermo Fisher, Waltham, MA, USA) prior to incubation with antibodies for the appropriate markers for 1 h at 4°C. The following fluorochrome-conjugated antibodies were used and were purchased from BioLegend (San Diego, CA, USA): for lineage markers CD3 CD19 CD20 CD56, allophycocyanin (APC); for CD80, phosphatidylethanolamine (PE); for CD86, BV450; for major histocompatibility complex II (MHCII), BV650; and for CD68, APC Cy7. For intracellular cytokine staining, brefeldin A was added to culture wells during the final cART treatment. Four hours later, cells were collected and stained with fluorochrome-conjugated antibodies purchased from BD Biosciences (San Jose, CA) as follows: for TNF-α, PerCP-Cy5.5; for IL-6, PE Texas Red; for IL-1β, fluorescein isothiocyanate (FITC); and for IFN-γ, APC. To assess cell proliferation, cells were stained for ki67 with PE Texas Red (BioLegend), a conventional marker for cell proliferation (83, 84). To measure cellular senescence markers, cells were stained for p53 with FITC (BioLegend) and p16 with PE (BD Biosciences, San Jose, CA). Stained cells were analyzed with a BD LSRFortessa flow cytometer (BD Biosciences). Data collected were plotted and quantified, using geometric mean fluorescence intensity (MFI), with FlowJo software version 10 (TreeStar, Ashland, Oregon).

### Senescence-associated β-galactosidase.

Measurement of SA β-galactosidase was performed using a fluorescence senescence assay kit (Abcam, Cambridge) per the manufacturer’s instructions. Briefly, the day after final cART or vehicle treatment, cells were incubated with senescence dye for 2 h at 37°C with 5% CO_2_. Cells were then washed and analyzed for fluorescence intensity (FL1) on a BD LSRFortessa flow cytometer.

### Cell cycle.

Flow cytometric analysis of cell cycle was performed on a BD LSRFortessa using CytoPhase Violet (BioLegend) per the manufacturer’s protocol. Briefly, at the end of the culture period, cells were incubated with 10 μM dye for 90 min at 37°C with 5% CO_2_. Cells were then washed and analyzed for fluorescence signal on a BD LSRFortessa flow cytometer.

### T cell proliferation.

CD45RA-positive (CD45RA^+^) naive T cells were isolated via negative selection (StemCell, Vancouver) according to the manufacturer’s protocol and cocultured with MDMs from each control and experimental group. Specifically, isolated naive T cells were labeled with CellTRace CFSE cell proliferation dye (Thermo Fisher) per the manufacturer’s instructions. Naive T cells were then activated with soluble anti-CD3 and anti-CD28 antibodies (BD Biosciences) at 1 μg/mL and then cocultured with donor-matched MDMs that were previously skewed toward M2a or left unskewed and each treated with Atripla, Triumeq, or vehicle control. After 72 h, cells were collected, and CFSE intensity was assessed by flow cytometry using a BD LSRFortessa flow cytometer.

### ROS/superoxide analysis.

For detection of ROS and superoxide, we used a ROS/superoxide detection assay kit (Abcam, Cambridge) per the manufacturer’s recommendations. Briefly, the green (ROS) and orange (superoxide) detection reagents were reconstituted in dimethylformamide (DMF), and then we prepared a ROS/superoxide detection mix by combining both reagents with culture medium. Cells were gently scraped and centrifuged at 400 × *g* for 5 min and then resuspended in fresh medium. For the negative controls, the cells were pretreated with *N*-acetyl-l-cysteine, an ROS inhibitor, and incubated for 30 min. Next, vehicle, cART, and the ROS inducer pyocyanin were added to the respective wells. The plates were then incubated for 1 h at 37°C with 5% CO_2_. Fluorescence intensity was measured for ROS (FL1) and for superoxide (FL2) on a BD LSRFortessa flow cytometer.

### MitoTracker Red.

Per the manufacturer’s instructions, MitoTracker Red staining was performed using a mitochondrial membrane potential staining kit (Molecular Probes, Eugene OR). Briefly, cells were harvested after cessation of cART treatment and staining with MitoTracker Red dye for 30 min at 37°C and 5% CO_2._ After staining, cells were washed and MitoTracker Red staining measured via flow cytometric analysis on PE Texas Red; fluorescent images were also captured using the Keyence All-in-One fluorescence microscope BZ-X800.

### Mito stress test.

Agilent Seahorse XF24 microplates, Seahorse XFe24 extracellular flux assay kits, and Seahorse XF cell Mito stress test kits were purchased from Agilent Technologies (Santa Clara, CA), and the protocol to assess mitochondrial dysfunction was performed following the manufacturer’s instructions (50). Freshly isolated monocytes were seeded in Seahorse XF24 microplates at the initiation of culture at a cell density of approximately 20,000 cells per well. Cells were immediately treated with M-CSF at day 0 (D0) and every other day until maturation at day 5. MDMs were then treated with either Triumeq, Atripla, or the individual components of each drug in duplicate wells for an additional 7 days. After treatment and the day before the Seahorse assay, the assay components were prepared per the manufacturer’s instructions. Briefly, assay calibrant was added to each well of the extracellular flux assay kit to hydrate the assay cartridges overnight. On the day of the assay, MDM cell culture medium was switched to complete Dulbecco’s modified Eagle medium (cDMEM) Seahorse assay medium and incubated at 37°C without CO_2_ for at least 45 min prior to the assay. Stock concentrations of oligomycin (100 μM), FCCP (100 μM), and rotenone and antimycin A (Rot/AA) (50 μM) were made using assay media and added at the indicated concentrations to their designated injection ports on the sensor cartridges to achieve final well volumes of 1.5 μM, 1 μM, and 0.5 μM, respectively. The assay was run using the Agilent Seahorse XFe24 analyzer. Data were exported and analyzed using Microsoft Office Excel 2013 Software and GraphPad Prism 5.

### RNA-seq following MDM maturation and treatment with cART.

Total RNA was purified using the miRNeasy kit (Qiagen, Hilden, Germany) per the manufacturer’s instructions and used for sequencing library construction. RNA samples were checked for purity using NanoDrop One spectrophotometer (Thermo Scientific) and analyzed for integrity using 4200 TapeStation (Agilent). Relative levels of remaining DNA were checked by dual RNA/DNA measurements using a Qubit fluorometer (Invitrogen). DNA amounts did not exceed 10% of the total amount of nucleic acids.

Sequencing libraries for Illumina sequencing were prepared using 50 ng of total RNA per sample. Library prep was carried out with the Universal Plus mRNA-Seq library preparation kit (Tecan/NuGen; catalog no. 0520-A01), as written in the product manual (NuGen; M01485 v5). In brief, RNA underwent poly(A) selection, enzymatic fragmentation, and generation of double-stranded cDNA using a mixture of oligo(dT) and random priming. The cDNA underwent end repair, ligation of dual-index adaptors, strand selection, and 14 cycles of PCR amplification. The number of cycles was determined by qPCR on a small aliquot of the unamplified libraries. All intermediate purification steps and final library purification were carried out using Agencourt AMPure XP beads (Beckman Coulter; catalog no. A63881).

Final amplified libraries were measured with the Qubit 1× double-stranded DNA (dsDNA) HS assay kit (Invitrogen; catalog no. Q33231), and fragment size distribution was confirmed to be approximately 470 bp using the D5000 ScreenTape assay (Agilent; catalog nos. 5067-5588 and 5067-5589).

The concentration of the final library pool was confirmed by qPCR and subjected to test sequencing in order to check sequencing efficiencies and accordingly adjust proportions of individual libraries. The pool was purified with the Agencourt AMPure XP beads (Beckman Coulter; catalog no. A63881), quantified by qPCR using KAPA library quantification kit, and sequenced on a NovaSeq 6000 S4 flow cell, 2 × 150 bp, approximately 30 M clusters per sample, at the University of Illinois Roy J. Carver Biotechnology Center High-Throughput Sequencing and Genotyping Unit.

### Bioinformatics analysis of RNA-seq.

Raw reads were trimmed to remove TruSeq adapters and bases from the 3′ end with quality scores less than 20 using Cutadapt (85); trimmed reads shorter than 40 bp were discarded. Trimmed reads were aligned reference genome hg38 using STAR (86). The expression level of Ensembl genes was quantified using FeatureCounts (87). Differential expression statistics were computed using edgeR (88, 89) on raw expression counts obtained from quantification. Donor IDs were used as batch factors in the generalized linear models (GLM) to control for individual-specific differences and in generating batch-corrected expression levels using the remove BatchEffect function. Normalized expression was computed as log_2_ counts per million (CPM), including a trehalose monomycolate (TMM) normalization and batch effect correction. Comparisons were made between drug treatments for each protein and between proteins for the control group. In all cases, *P* values were adjusted for multiple testing using the false-discovery rate (FDR) correction of Benjamini and Hochberg (90). Heatmaps and volcano plots were also generated within the R programming language. An absolute log fold change (logFC) value of greater than 2 and *q* value of less than 0.05 were used to generate the volcano plots. Clustering was performed using k-means clustering, selecting the number of clusters (*k*) with reproducibility clustering statistics, similar to the approach outlined by Senbabaoglu and coworkers (91). K-means clustering was performed with 10 random initializations on a range of cluster numbers *k* (2 to 20). For each *k*, the reproducibility of the repeated clustering runs was computed by comparing the pairwise distance between clustering results as the number of coclustered feature pairs shared between results divided by the number of coclustered feature pairs within each result individually. This difference was averaged across all result pairs for each value of *k*, and the largest *k* with an average distance less than 1e-5 will be selected as the optimal *k* with highly reproducible clusters. Clustering analysis was run separately for all differentially expressed genes (DEGs) due to treatments with M-CSF to generate M-MDMs. Z-scored log-scaled normalized expression for each cluster was visualized using heat maps and boxplots to aid in interpretation. Gene sets obtained from the clustering analysis were run through the core analysis function in ingenuity pathway analysis (IPA). Enriched canonical pathways and upstream regulators were determined based on the Benjamini Hochberg corrected *P* value from IPA. Enrichment clusters for features that were significant (*q* < 0.05) were generated using Cytoscape (92). Venn diagrams were also generated using an open-source Venn diagram viewer (93).

### Statistics.

Statistical analyses were performed using Graph Pad Prism version 5 (San Diego, CA). Student’s *t* tests were used for comparisons between two groups, and one-way analyses of variance (ANOVAs) and two-way ANOVAs were used for three of more groups with one and two variables, respectively. Error bars show plus or minus standard error of the mean (SEM) and *P* values of less than 0.05 were considered to be statistically significant.

### Data availability.

RNA-seq data are available in the Gene Expression Omnibus (GEO) under accession number GSE195708.
