# Supplementary material for: Anti-HIV Drugs Cause Mitochondrial Dysfunction in Monocyte-Derived Macrophages
Source: Antimicrob Agents Chemother. 2022 Mar 16;66(4):e01941-21. doi: 10.1128/aac.01941-21 (PMC9017340; doi:10.1128/aac.01941-21)
Supplement: SUPPLEMENTAL FILE 4 — Supplemental figures. Download aac.01941-21-s0001.pdf, PDF file, 0.2 MB [file aac.01941-21-s0001.pdf]

Supplementary Figures

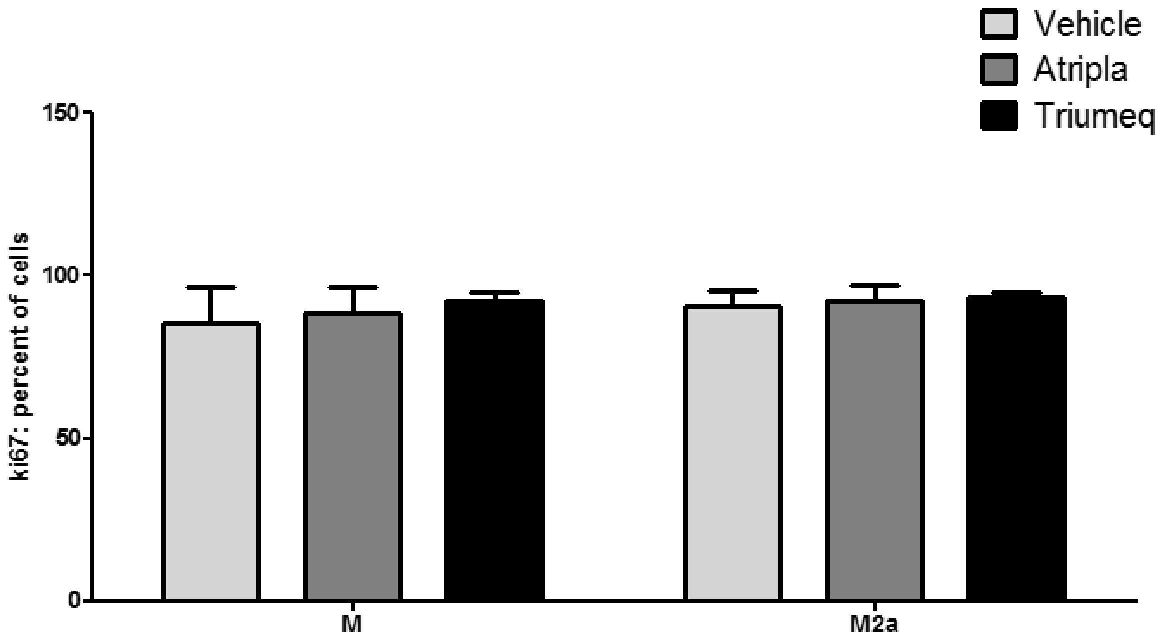

Fig S1. Ki67 expression on cART treated MDMs. Cells were stained with a fluorescence antibody against ki67 and signal measured by flow cytometry and represented as percentage.

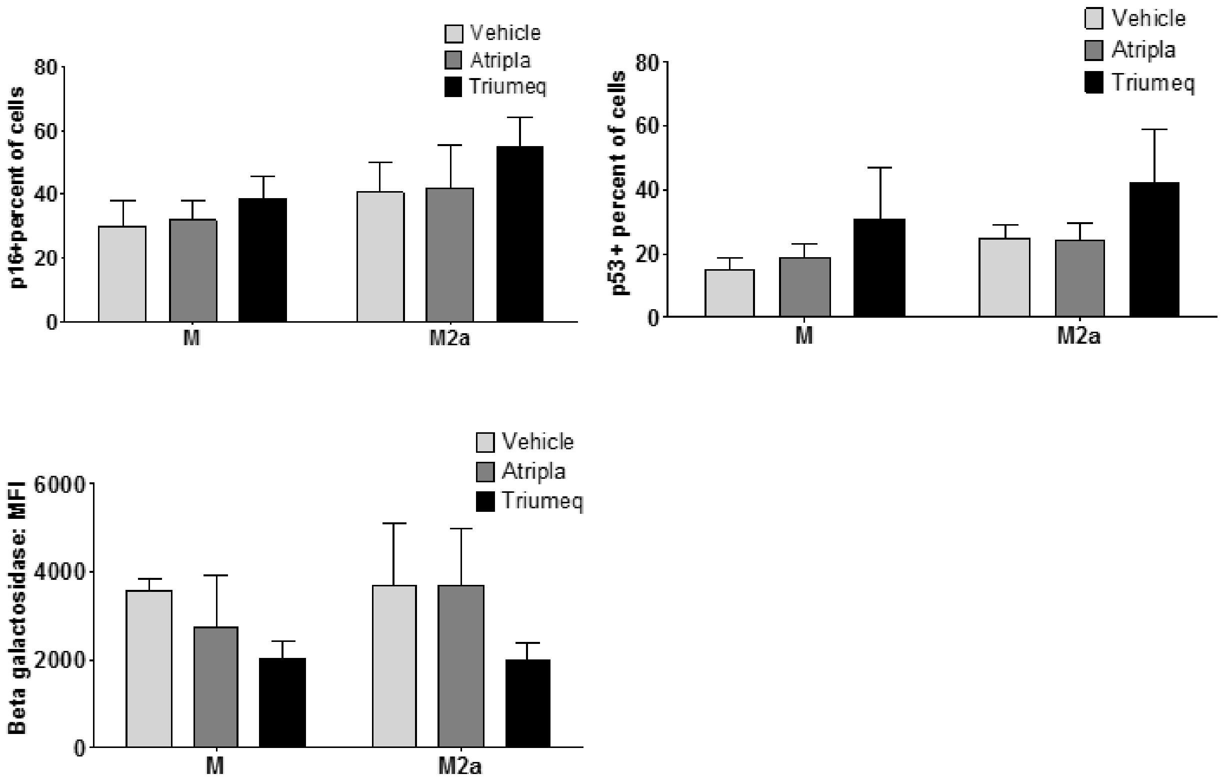

Fig. S2. cART has no effect on the expression of senescent markers. Senescent markers A. p16 and B. p53 were assessed via intracellular staining and flow cytometry and represented as MFI. **B.** A fluorescent senescence assay kit was used to detect β-galactosidase in the culture wells the day after final cART and vehicle treatment. Signal was measured via flow cytometry on AF488. Two-way ANOVA, n=3. MFI: (geometric) mean fluorescence intensity, AF488: Alexa Fluor 488.
